# Simultaneous Determination of Steroids and NSAIDs, Using DLLME-SFO Extraction and HPLC Analysis, in Milk and Eggs Collected from Rural Roma Communities in Transylvania, Romania

**DOI:** 10.3390/molecules29010096

**Published:** 2023-12-22

**Authors:** Mihaela Cătălina Herghelegiu, Vlad Alexandru Pănescu, Victor Bocoș-Bințințan, Radu-Tudor Coman, Vidar Berg, Jan Ludvig Lyche, Maria Concetta Bruzzoniti, Mihail Simion Beldean-Galea

**Affiliations:** 1 Faculty of Environmental Science and Engineering, Babeș-Bolyai University, 1 Kogălniceanu Str., 400084 Cluj-Napoca, Romania; 2 Faculty of Medicine, “Iuliu Hațieganu” University of Medicine and Pharmacy, 8 Babeș Str., 400012 Cluj-Napoca, Romania; 3 Faculty of Veterinary Medicine, Norwegian University of Life Sciences, 1433 Ås-Oslo, Norway; 4 Department of Chemistry, University of Turin, Via P. Giuria 5, 10125 Turin, Italy; mariaconcetta.bruzzoniti@unito.it

**Keywords:** steroids, non-steroidal anti-inflammatory drugs (NSAIDs), dispersive liquid–liquid microextraction based on solidification of floating organic droplets (DLLME-SFO), milk, eggs, rural Roma communities

## Abstract

This research aims to determine five steroids and four non-steroidal anti-inflammatory drugs in milk and egg samples collected from rural Roma communities in Transylvania, Romania. Target compounds were extracted from selected matrices by protein precipitation, followed by extract purification by dispersive liquid–liquid microextraction based on solidification of floating organic droplets. The extraction procedure was optimized using a 2^4^ full factorial experimental design. Good enrichment factors (87.64–122.07 milk; 26.97–38.72 eggs), extraction recovery (74.49–103.76% milk; 75.64–108.60% eggs), and clean-up of the sample were obtained. The method detection limits were 0.74–1.77 µg/L for milk and 2.39–6.02 µg/kg for eggs, while the method quantification limits were 2.29–5.46 µg/L for milk and 7.38–18.65 µg/kg for eggs. The steroid concentration in milk samples was <MDL up to 4.30 µg/L, decreasing from 17α-ethinyl estradiol, 17β-estradiol, and estrone to estriol. The NSAID concentration was <MDL up to 3.41 µg/L, decreasing from ibuprofen, diclofenac, and ketoprofen to naproxen. The steroid concentration in the egg samples was <MDL to 2.79 µg/kg, with all steroids detected, while the concentration of NSAIDs was <MDL to 2.28 µg/kg, with only ibuprofen, ketoprofen, and naproxen detected. The developed protocol was successfully applied to the analysis of target compounds in real milk and egg samples.

## 1. Introduction

Milk and eggs are an indispensable part of the daily diet of individuals, as they contain the necessary nutrients (high-quality proteins, carbohydrates, vitamins, trace minerals) for human health [1,2]. These two foods are widely consumed, especially by growing children and elderly people, since they make a valuable contribution to the nutritional balance of the diet and overall are a relatively inexpensive source of nutrients [1,3].

However, currently, a range of veterinary drugs are widely used in animal husbandry both to prevent and treat diseases; they may also be used as growth promoters by direct application or mixed with feed [4,5].

As a result of the abuse of veterinary products or through their improper use, non-respected withdrawal periods, and cross-contamination, these drugs can be transferred and may accumulate in products of animal origin, which is definitely a potential risk to public health [4,5,6]. Non-steroidal anti-inflammatory drugs (NSAIDs) are widely used in veterinary medicine to treat inflammatory processes [7]. In order to protect consumers’ health, the European Union established threshold values called “maximum residue limits” (MRLs) in food of animal origin; for instance, that for diclofenac MRL is just 0.1 μg/kg [4,8]. Regarding the steroid hormones, the European Union banned their use as animal growth promoters [9,10], but despite this prohibition, there have been no specific maximum residue limits established [11]. Steroid hormones (17-β-estradiol or its ester-like derivatives) can only be used for the treatment of cattle in the case of fetus maceration or mummification and for pyometra (serious infection of the uterus) [10,12]. Glucocorticoids (like hydrocortisone) are used in the animal husbandry industry due to their anti-inflammatory and immunosuppressive properties [13]. Natural steroid hormones (the endoestrogens estrone, estradiol, and estriol) are natural substances that strongly influence female characteristics and are normally present in milk and eggs [12,14], while synthetic hormones (the exoestrogens, such as 17α-ethinylestradiol) are used to improve the weight gain of animals [12]. Human health can be affected (hormonal disorders and diseases) by food when estrogen levels are higher than normal [11] or, in the case of hydrocortisone, athletes can test positive for doping [13].

The low concentrations at which NSAIDs and estrogens are present in milk and eggs require a suitable method of extraction. A few scientists have already used liquid–liquid extraction (LLE) [14,15,16] and solid-phase extraction (SPE) [17] to isolate and concentrate NSAIDs and estrogens from milk and eggs. These two extraction techniques require, however, a relatively high consumption of solvent on one hand, and a number of purification steps that are time-consuming on the other.

To overcome these problems, over time, miniaturized extraction methods have been developed for both the solid phase [18] and the liquid phase [19], with the aim of reducing the volume and toxicity of the extraction solvent and the amount of sample being processed to reduce the extraction time [20]. Due to the advantages offered, their applications are numerous [21], which is why we only mention their applicability to the analysis of steroids in urine [22] and in water [23]; of NSAIDs in water [24]; of steroids and NSAIDs in wastewater [25]; and, last but not least, in the analysis of steroids and NSAIDs in food samples [5,26,27,28,29]. These are the target compounds selected for this study. These microextraction methods are faster, more environmentally friendly, and have higher enrichment factors [30].

The main aim of this study was to develop a dispersive liquid–liquid microextraction based on solidification of the floating organic droplet (DLLME-SFO) method, followed by HPLC-PDA analysis for the simultaneous determination of four NSAIDs (naproxen, ketoprofen, diclofenac, and ibuprofen), four estrogens (estrone, 17β-estradiol, 17α-ethinylestradiol, and estriol), and one glucocorticoid (hydrocortisone) in both bovine milk and chicken eggs. The developed DLLME-SFO–HPLC-PDA method was then successfully applied to the analysis of these drugs in bovine milk and chicken egg samples collected from households and local markets in 15 rural Roma communities in Transylvania, Romania.

## 2. Results and Discussion

### 2.1. Optimization of DLLME-SFO Conditions

Based on our previously published work [25], we choose 1-undecanol as the most suitable solvent for the extraction of the selected compounds and acetonitrile as the most suitable dispersion solvent.

For the optimization of DLLME-SFO, a water sample with a volume of 10 mL was spiked with 100 ng of each compound and then subjected to the extraction protocol under different extraction conditions. The extraction efficiency was expressed by relative extraction recovery of the selected compounds from each experiment, and was used to optimize the extraction conditions.

A total of sixteen experiments using different values of the four variables set at minimum (−1) and maximum (+1) values, and three experiments at the medium set point (0), were carried out. The obtained extraction recovery for each experiment is presented in the Supplementary Materials (Table S1).

The data obtained in the experiments were subjected to optimization using desirability functions (Supplementary Materials, Figure S1).

The maximum, minimum, and optimum extraction recoveries for each compound are presented in Table 1.

The mathematical relationship between the response *Y* (enrichment recovery) and the four independent variables, *X*_1_, *X*_2_, *X*_3_, and *X*_4_ can be modeled by a linear polynomial equation [31] including four linear terms, six two-way interaction terms, four three-way interaction terms, one four-way interaction term, and one intercept term, as follows:

*Y* = *β*_0_ + *β*_1_*X*_1_ + *β*_2_*X*_2_ + *β*_3_*X*_3_ + *β*_4_*X*_4_ + *β*_12_*X*_1_*X*_2_ + *β*_13_*X*_1_*X*_3_ + *β*_14_*X*_1_*X*_4_ + *β*_23_*X*_2_*X*_3_ + *β*_24_*X*_2_*X*_4_
+ *β*_34_*X*_3_*X*_4_ + *β*_123_*X*_1_*X*_2_*X*_3_+ *β*_124_*X*_1_*X*_2_*X*_4_ + *β*_134_*X*_1_*X*_3_*X*_4_ + *β*_234_*X*_2_*X*_3_*X*_4_ + *β*_1234_*X*_1_*X*_2_*X*_3_*X*_4_ (1)

where *β*_0_ is a constant; *β*_1_, *β*_2_, *β*_3_, and *β*_4_ are linear coefficients; and *β*_12_, *β*_13_, *β*_14_, *β*_23_, *β*_24_, *β*_34_, *β*_123_, *β*_124_, *β*_13_, *β*_234_, and *β*_1234_ are interaction coefficients.

For the present study, taking diclofenac as an example, the empirical relationship between the independent variables (*X*_1,_ *X*_2,_ *X*_3_, *X*_4_) and the response (*Y*) can be represented by the following equation:

*Y* = 74.6 + 2.00*X*_1_ + 0.65*X*_2_ + 10.99*X*_3_ + 1.70*X*_4_ + 0.98*X*_1_*X*_2_ + 0.11*X*_1_*X*_3_ − 3.24*X*_1_*X*_4_
− 0.98*X*_2_*X*_3_ − 0.64*X*_2_*X*_4_ − 3.38*X*_3_*X*_4_ + 0.39*X*_1_*X*_2_*X*_3_ + 0.35*X*_1_*X*_2_*X*_4_ − 1.53*X*_1_*X*_3_*X*_4_ − 1.28*X*_2_*X*_3_*X*_4_ + 2.11*X*_1_*X*_2_*X*_3_*X*_4_ (2)

The linear polynomial equation showed that the four linear coefficients had a positive effect. However, this positive effect was counterbalanced by the negative effects given by the interactions between two and three combined variables. The magnitude of these effects can be better seen in the histogram (Table S2) that includes the significance levels of the factors and their interactions.

It is important to specify that the magnitude of the influence of these factors is essentially related to the significance range used for each variable in the experimental design, since coded values were used for optimization.

The optimum conditions predicted by the model were, in coded values: +1 for salt, +0.518 for disperser volume, +0.518 for extraction solvent volume, and −0.497 for pH (Supplementary Materials Figure S1), which correspond to the following experimental conditions: salt (NaCl) amount (ionic strength): 500 mg, dispersion solvent volume (acetonitrile): 239.75 μL, extraction solvent volume (1-undecanol): 85.54 μL, and sample pH: 3.51. For a better measurement, we used the following values: amount of salt (NaCl): 500 mg, volume of dispersion solvent (acetonitrile): 240 μL, volume of extraction solvent (1-undecanol): 85 μL; and pH of the sample: 3.5.

The previously presented conditions are optimal theoretical conditions; therefore, to be used in subsequent experiments, they must be experimentally validated. This step is necessary because the chosen experimental design model is quite simple, and consequently, its predictive capabilities could be limited.

### 2.2. Validation of DLLME-SFO-LC-PDA Method

The performance of the developed DLLME-SFO was expressed in terms of accuracy, intra- and inter-day precision, linearity, limit of detection (LOD), limit of quantification (LOQ), extraction recovery (ER), and enrichment factor (EF).

*The linearity* of the method was studied in a concentration range between 1.5 and 25 ng/mL. For this, different standard mixtures were prepared at concentrations of 1.5, 3.0, 6.25, 12.5, and 25 ng/mL of each compound by successive dilution of the stock solution. After the HPLC analysis, calibration curves were constructed for each compound by graphically representing the peak area as a function of the analyte concentration. Good linearities were obtained for all compounds, with correlation coefficients (R^2^) greater than 0.99 (Table 2).

*The instrument limit of detection* (*LOD*) *and limit of quantification* (*LOQ*) were determined by statistical approaches using the standard deviation (SD) of the regression line and the slope (S) of each calibration curve.

The instrument limits of detection were between 0.07 and 0.21 ng/mL for steroids and between 0.8 and 0.12 ng/mL for NSAIDs; the instrument limits of quantification were between 0.22 and 0.65 ng/mL for steroids and between 0.24 and 0.35 ng/mL for NSAIDs (Table 2).

*Accuracy* was expressed by the extraction recovery of analytes from the spiked water sample. To this end, the optimal DLLME-SFO parameters predicted by the model were experimentally validated. In this regard, a volume of 10 mL of Milli-Q water was spiked with 100 ng of each selected compound, then acidified to pH 3.5. This was followed by the addition of 500 mg of NaCl, 85 μL of 1-undecanol as extraction solvent, and 240 μL acetonitrile as disperser, as well as centrifugation for 4 min at 4500 rpm. The obtained results were between 74.00 and 111.6% for all selected compounds, except hydrocortisone, for which the recovery was 14.99% (Table 2).

*Intra- and inter-day precision* were evaluated by relative standard deviations (RSD%) on Milli-Q water samples spiked with 100 ng of each compound. The obtained RSD% values were below 3.81% for the intra-day precision and below 3.84% for the inter-day precision for all the compounds tested (Table 2), thus agreeing with the requirements of the method’s validation procedures regarding the compounds in the range of concentration of the order of μg/L.

*Enrichment factors* were calculated considering the ratio of analyte concentration in the collected organic phase and the initial concentration of the analyte in the liquid sample. The results showed that the developed DLLME-SFO protocol provided an EF between 87.1 and 131.3, except for hydrocortisone, for which the EF was 17.64 (Table 2), comparable to classical solvent extraction [15,16].

### 2.3. Matrix Effect

To study the matrix effect, 100 ng of each selected compound was added to 10 mL of the milk sample and to 3 g of the homogenized egg sample, which was diluted to 10 g with double-distilled water. The resulting samples were subjected to deproteinization with 10 mL of ACN:acetic acid mixture (95:5, *v*/*v*) and 20 g of NaCl [32,33]. Afterwards, the samples were centrifuged at 4000 rpm for 5 min, after which the supernatant was collected and further cleared by defatting with 4 mL of n-hexane [12]. After the removal of the hexane layer, the remaining phase was evaporated to dryness, and the residue was reconstituted in 10 mL double-distilled water for both the milk and egg samples. The two resulting samples were subjected to the previously described DLLME-SFO protocol. All experiments were performed three times (Table S3). Method detection limits (MDL) and method quantification limits (MQL) were obtained by dividing the LOD and LOQ by the enrichment factors obtained for the milk and egg samples. The performances of the developed DLLME-SFO are presented in Table 3.

As shown in Table 3, the developed DLLME-SFO protocol provided good extraction recovery, enrichment factors, and method detection and quantification limits (MDL, MQL).

The *extraction recoveries* of the target compounds exceeded 80% in both the milk and egg samples, except for hydrocortisone, for which the ER was below 15%. For estriol (E3), the obtained ER varied between 74.49% for the milk samples and 75.64% for the egg samples, but, considering the quantity of the order of ng, this can be considered satisfactory. Moreover, the ER values for the milk and egg samples were comparable to the values obtained for the contaminated water samples (Table 2) suggesting that no matrix effect occurred for the milk and egg samples.

The *enrichment factor* (EF) for milk samples ranged between 87.64 and 122.07, while the EF for milk samples was between 26.97 and 38.72, with the unique exception of hydrocortisone, for which the EF was 14.49 for milk samples and 4.32 for egg samples.

The *MDL* varied between 0.74 and 1.77 µg/L for milk samples and between 2.39 and 6.02 µg/kg for egg samples.

The *MQL* varied between 2.29 and 5.46 µg/L for milk samples and between 7.38 and 18.65 µg/kg for egg samples. For hydrocortisone, the MDL and MQL were on the order of tens of µg/L and µg/kg, but considering the low ER, this cannot be used as a real value for practical application.

**Figure 1:**
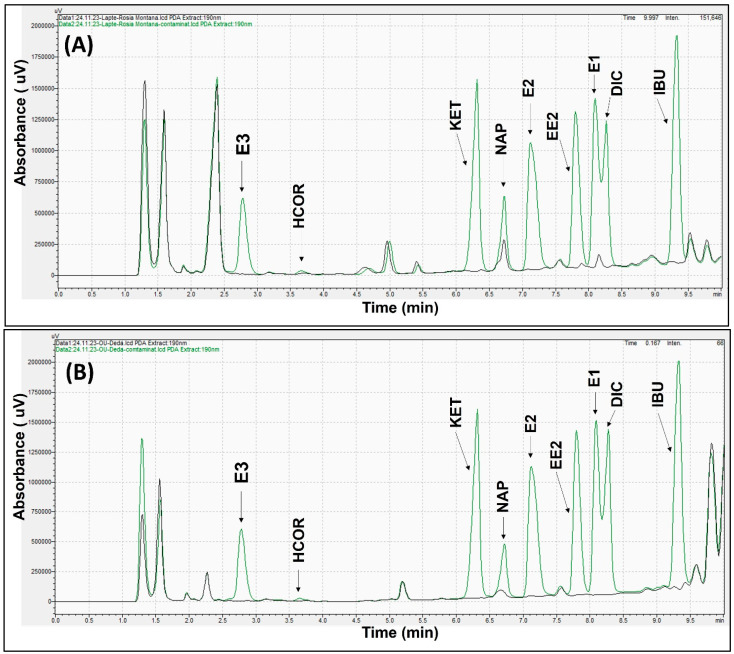
Chromatograms obtained at 190 nm of milk sample ((**A**) black color) and spiked milk sample with 100 ng of each compound ((**A**) green color). Chromatograms of egg sample ((**B**) black color) and spiked egg sample with 100 ng of each compound ((**B**) green color).

Furthermore, as demonstrated in Figure 1A,B, the extraction protocol provided good sample cleanup, with no other peaks interfering with the target compounds.

In conclusion, the developed protocol can be used for the analysis of target compounds in real milk and egg samples, meeting all the requirements for this type of analysis.

### 2.4. Analysis of Milk and Egg Samples Collected in Rural Roma Communities

After studying the matrix effect, the developed protocol was applied to the analysis of milk and egg samples collected from households and local markets in 15 rural Roma communities in Transylvania, Romania. A total of 15 milk samples and 15 egg samples were analyzed.

The results showed that, in the milk samples, steroids were found in 10 samples in concentrations that varied from <MDL to 4.30 µg/L; EE2 was found in 8 samples; and E2, E1, and E3 were found in just 3 samples. NSAIDs were found in seven samples in concentrations ranging from <MDL to 3.41 µg/L; their occurrence was as follows: ibuprofen was found in seven samples; diclofenac in three samples; ketoprofen in two samples, and naproxen in one sample. The results obtained for each compound in the analyzed milk samples are presented succinctly in the Supplementary Materials (Table S4).

In the egg samples, steroids were found in 13 samples in concentrations ranging from <MDL to 2.79 µg/kg; they ranked as follows: E3 (found in 7 samples), E2 (in 5 samples), EE2 (in 4 samples), and E1 (in just 1 sample). NSAIDs were found in 12 samples in concentrations that ranged from <MDL to 2.28 µg/kg; their occurrence was as follows: ibuprofen (found in 8 samples), naproxen (in 7 samples), and ketoprofen (in 6 samples). It is worth mentioning that diclofenac was not detected in any egg samples. The results obtained for each compound in the egg samples are briefly presented in the Supplementary Materials (Table S5).

If the concentrations of these target compounds in the analyzed milk samples are to be compared with other results obtained worldwide, one will notice that the concentrations of steroids obtained in this work were higher than those of US milk samples (estrone: 23–67 ng/L, estradiol: <10 ng/L) [34], but in the same concentration range as milk samples from Brazil (estradiol: 86.54–171.09 µg/L) [35] and China (0.05–3.2 µg/kg) [33].

The concentrations of steroids found in the egg samples were in the same range as samples from Spain (estrone: 0.48–1.7 µg/kg and 17-B estradiol: 1.7–2.7 µg/kg) [32] and China (estrone: 0.05–1.72 µg/kg; 17α-estradiol: <LOD—0.13 µg/kg; 17β-estradiol: <LOD—0.16 µg/kg [36]; and estrone: 0.12–0.84 µg/kg) [15].

### 2.5. Comparison with Other Reports

To highlight the benefits of the developed method, its performance was compared with the performances of other developed methods (Table 4). These performances were evaluated in terms of the amount of sample, volume of extraction solvent, LOQ, and ER.

By analyzing the data shown in Table 4, we can conclude that the developed method has a performance comparable to or better than other methods used for the analysis of NSAIDs and hormones in milk and egg samples. Thus, the method has an LOQ in the range of µg/L µg/kg and a recovery of over 80%, and requires a small amount of the sample and extraction solvent volume. Another advantage is the fact that both NSAIDs and steroids are extracted and analyzed together. We can, therefore, state that the method meets all the requirements for a green method of extraction and analysis.

## 3. Material and Methods

### 3.1. Chemicals and Reagents

For the proposed experiments, four NSAIDs, namely, diclofenac sodium salt (DIC), ibuprofen (IBU), ketoprofen (KET), and naproxen (NAP), together with four estrogens, namely, estrone (E1), 17β-estradiol (E2), 17α-ethynylestradiol (EE2), and estriol (E3), and one glucocorticoid, namely, hydrocortisone (HCOR), all with purity levels >98%, were purchased from Sigma-Aldrich (Steinheim, Germany). The molecular structure and some physico-chemical properties of all target compounds are presented in the Supplementary Materials (Table S6).

Stock standard solutions of the individual compounds, at a concentration of 1000 mg/L each, were prepared in acetonitrile for ibuprofen, ketoprofen, and naproxen, while diclofenac and the hormones were prepared in methanol. All prepared stock standards were stored at 4 °C in the dark until analysis. Acetonitrile and methanol of HPLC grade, 1-undecanol, NaCl, ortho-phosphoric acid, acetic acid, and *n*-hexane were all purchased from Merck (Darmstadt, Germany).

Milli-Q water was prepared using a Milli-Q-Plus ultrapure water system (Millipore, Milford, MA, USA). An Eppendorf centrifuge, model 5804 R (Eppendorf, Wien, Austria), was used for the centrifugation of the samples.

### 3.2. Instrumentation

HPLC analyses were carried out using Shimadzu equipment (SLC-40D) with a photodiode detector (SPD-M40). Instrument control and data acquisition were carried out via LabSolution software (Version 5.101). The separation of the target compounds was performed on a ZORBAX Eclipse Plus C18 (150 × 4.6 mm, 5 μm) column with a flow rate of 1.0 mL/min and an oven temperature of 40 °C. The injection volume was 20 µL. The gradient elution (acetonitrile ACN and KH_2_PO_4_ 25 mM) program which was employed was 35% ACN, maintained for 2 min, then increased to 80% in 8 min and held for 5 min, then finally decreased to 35% ACN within 5 min.

The specific UV wavelengths used for PDA detection were as follows: ketoprofen—256 nm, naproxen—230 nm, diclofenac—200 nm, ibuprofen—190 nm, 17β-estradiol—200 nm, 17α-ethynylestradiol—195 nm, estrone—195 nm, estriol—197 nm, and hydrocortisone—247 nm. These UV wavelengths were established by UV spectra generated by the SPD detector for each single compound.

### 3.3. Extraction by DLLME-SFO Protocol

For the extraction procedure, 10 g of milk was weighed into a 50 mL polypropylene centrifuge tube. The eggs were shaken to mix the white and yolk, after which 3 g of the mixture was weighed and homogenized with 7 g of distilled water. For deproteinization, 10 mL of ACN:acetic acid mixture (95:5, *v*/*v*) and 2 g of NaCl were added to each tube and vortexed, followed by centrifugation at 4000 rpm for 5 min [32,33]. Afterwards, 8.5 mL of the supernatant was collected and further cleared by defatting with 4 mL of n-hexane [12]. The n-hexane phase was removed, and the acetonitrile phase was then evaporated to dryness. The resulting residues were reconstituted in 10 mL of water acidified at pH 3.5 with 10 µL 10% ortho-phosphoric acid solution, after which 0.500 g of NaCl was added, followed by the addition of 325 µL of extraction mixture containing 240 µL of ACN and 85 µL of 1-undecanol. After centrifugation at 4000 rpm for 5 min to separate the two phases, the tube was cooled in an ice-water bath for 15 min to solidify the 1-undecanol, which was then collected with a spatula and transferred to a conical ampoule [25]. After melting the extract at room temperature, a volume of 20 μL was directly injected into the HPLC for analysis.

### 3.4. Enrichment Factor and Extraction Recovery

DLLME-SFO efficiency was expressed by the enrichment factor (EF) and relative extraction recovery (ER%) using the following equations [37]:

(3) $$EF=\frac{C_{col}}{C_{aq}}$$

(4) $$ER\%=\frac{\left(n_{col}\right)}{\left(n_{aq}\right)}\times 100=\frac{C_{col}\times V_{col}}{C_{aq}\times V_{aq}}\times 100$$

where C_col_ is the analyte’s concentration in the collected organic phase; C_aq_ is the initial concentration of the analyte in the liquid sample; n_col_ is the total amount of analyte extracted in the collected organic phase; n_aq_ is the total amount of analyte in the liquid sample; and V_col_ and V_aq_ represent the volume of the collected organic phase and the volume of the liquid sample, respectively.

To achieve good efficiency of the extraction method, the extraction recovery must be between 80% and 120% [38].

### 3.5. Statistical Approach Used for DLLME-SFO Optimization

Optimizing the experimental conditions is a stage that requires a large number of experiments, which in turn depends on the number of variables (factors) that can affect the experimental results. Usually, the optimal conditions are determined by univariate approaches that consist of changing one variable and observing the effects, while the other variables are kept constant [39]. However, this approach is time-consuming and does not describe the potential interactions between the variables.

A very efficient way to minimize the number of experiments is the statistical approach, such as a multivariate experimental design strategy. This approach requires fewer experiments and can also be used for quantitative and qualitative modeling of relationships between factors and responses, with the goal of maximizing the extraction efficiency [40].

In the present study, a 2^4^ experimental design with a triplicate center point (0) was chosen, and each variable was investigated at two levels (−1 and +1). The studied variables were the amount of sodium chloride (X1), the volume of the dispersant (X2), the volume of the extraction solvent (X3), and the pH of the sample (X4). The extraction efficiency was expressed by extraction recovery. The minimum values (−1), the center point (0), and the maximum values (+1) for each variable are presented in Table 5.

The desirability approach was used for the simultaneous optimization of variables to achieve maximum extraction recovery [41,42]. To this end, better individual desirability (value 1) corresponded to higher extraction recovery, while poor individual desirability (value 0) corresponded to zero extraction recovery, with a linear variation between these two values. Finally, the individual desirability of each compound contributed the same weight to the overall desirability function.

For all statistical modeling, the JMP14 (SAS Institute, Cary, NC, USA) statistical software package was used.

### 3.6. Sample Collection

For the analysis of the selected compounds (NSAIDs and steroids), samples of fresh cow’s milk and chicken eggs were collected from the local market and from local producers in 15 different rural Roma communities in the Transylvania region of Romania between June and November 2022. The spatial distribution of the sampling points is shown in the Supplementary Materials (Figure S2). Immediately after collection, the milk samples were frozen, and the egg samples were homogenized and stored in the freezer until analysis.

## 4. Conclusions

A simple, fast, and cheap DLLME-SFO procedure for the simultaneous extraction and preconcentration of NSAIDs and hormones in milk and egg samples was developed, optimized, validated, and finally tested using real food samples.

The target compounds (four estrogenic hormones + four NSAIDs + one corticoid) were extracted from milk and egg matrices after protein precipitation, followed by extract purification by DLLME-SFO.

The extraction procedure was optimized prior using a 2^4^ full factorial experimental design.

Good enrichment factors (87.64–122.07 for milk and 26.97–38.72 for eggs) and extraction recovery values (74.49–103.76% for milk; 75.64–108.60% for eggs) were obtained.

The clean-up of the sample also translated into a simpler chromatographic response that was easier to interpret.

The method detection limits were between 0.74 and 1.77 µg/L for milk and between 2.39 and 6.02 µg/kg for eggs, while the method quantification limits were between 2.29 and 5.46 µg/L for milk and between 7.38 and 18.65 µg/kg for eggs.

Steroid concentrations in milk samples ranged from <MDL to 4.30 µg/L; these were found in decreasing concentrations in 17α-ethinyl estradiol, 17β-estradiol, estrone, and estriol.

The concentration of NSAIDs in the milk samples ranged from <MDL to 3.41 µg/L, and decreased in the order of ibuprofen > diclofenac > ketoprofen > naproxen.

The concentration of steroids in the egg samples ranged from <MDL to 2.79 µg/kg for all steroids detected.

The concentration of NSAIDs in egg samples ranged from <MDL to 2.28 µg/kg, with only ibuprofen, ketoprofen, and naproxen detected. Diclofenac was not found in any egg sample.

Fast analysis was achieved, with an DSSME-SFO extraction performed in ca. 20 min, followed by a chromatographic separation step performed in less than 10 min. The specificity was further enhanced by using different UV detection wavelengths for each target analyte.

Our experimental findings are consistent, in an excellent manner, with the experimental work achieved worldwide so far.

Finally, we can mention that the DLLME-SFO procedure proved its applicability to the analysis of real milk and egg samples, and could open the way to a suitable and green alternative to the traditional SPE and LLE techniques for sample preparation in terms of performance and speed.

## Figures and Tables

**Table 1:** Maximum, minimum, and optimum extraction recoveries obtained by simulation.

Compound	Abbrev.	Extraction Recovery %		
		Minimum	Maximum	Optimum
Hydrocortisone	HCOR	15.25	32.03	23.64
Estrone	E1	42.50	96.77	69.64
17-β estradiol	E2	60.79	107.96	84.37
17-α ethynilestradiol	EE2	76.93	89.37	83.16
Estriol	E3	45.09	87.97	66.53
Ketoprofen	KET	48.99	114.09	81.54
Naproxen	NAP	51.92	117.85	84.88
Ibuprofen	IBU	72.31	98.34	85.32
Diclofenac	DIC	69.86	100.22	85.04

**Table 2:** Figure of merits: linearity, correlation coefficient (R^2^), LOD, LOQ, intra-day precision, inter-day precision, extraction recovery (ER), and enrichment factor (EF).

Compound	Calibration Curve Data			LOD (µg/mL)	LOQ (µg/mL)	Precision [RSD%]		ER [%]	EF
	Slope	SD	R²			Intra-	Inter-		
HCOR	42,454	1145.5	1	0.09	0.27	3.13	2.83	14.99	17.64
E1	148,521	9617.5	0.9993	0.21	0.65	3.35	3.65	110.5	130.1
E2	143,601	4070.9	0.9998	0.09	0.28	2.55	2.91	111.6	130.0
EE2	143,741	4473.1	0.9975	0.10	0.31	2.56	2.87	110.5	131.3
E3	117,929	2612.2	0.9999	0.07	0.22	3.70	3.35	74.0	87.1
KET	76,407	2193.3	0.9999	0.09	0.29	2.73	3.26	89.3	105.0
NAP	304,120	7921.0	0.9998	0.09	0.26	2.19	3.10	93.2	109.6
DIC	149,579	3557.8	0.9999	0.08	0.24	3.81	2.91	85.1	100.2
IBU	244,108	8562.2	0.9988	0.12	0.35	3.65	3.84	111.2	130.8

**Table 3:** The performances of the developed DLLME-SFO for milk and egg samples.

Compound	Milk Amount (ng) *		Egg Amount (ng) **		ER (%)		EF		Milk (µg/L)		Egg (µg/kg)	
	Initial	Found	Initial	Found	Milk	Egg	Milk	Egg	MDL	MQL	MDL	MQL
HCOR	nd	10.47	nd	10.31	12.32	12.13	14.49	4.32	6.21	18.63	20.83	62.50
E1	0.11	86.06	0.28	83.40	101.12	97.79	118.96	34.86	1.77	5.46	6.02	18.65
E2	0.06	88.26	0.26	89.92	103.76	105.48	122.07	37.60	0.74	2.29	2.39	7.45
EE2	1.17	83.05	nd	88.89	96.32	104.58	113.32	37.28	0.88	2.74	2.68	8.32
E3	0.08	63.32	0.35	64.29	74.49	75.64	87.64	26.97	0.80	2.51	2.60	8.16
KET	nd	77.86	0.17	76.08	91.60	89.31	107.76	31.84	0.84	2.69	2.83	9.11
NAP	0.40	74.40	0.82	76.70	87.06	89.27	102.42	31.83	0.88	2.54	2.83	8.17
DIC	0.07	74.58	0.12	77.63	87.66	91.19	103.13	32.51	0.78	2.33	2.46	7.38
IBU	0.30	87.73	1.57	93.88	102.86	108.60	121.01	38.72	0.99	2.89	3.10	9.04

* Standard deviation range: 0.34 to 1.72; ** standard deviation range: 0.47 to 2.27a; “nd”: not detected.

**Table 4:** Comparison of the proposed method with other reports.

Analyzed Compounds	Method	Matrix	Sample Amount	Extractant	LOQ	ER (%)	Ref.
***Hormones***							
10 steroids	DLLME	urine	3.0 mL	Cloroform	0.25–10 µg/L	70–90%	[22]
9 steroids	DLLME-SFOD	water	5 mL	2-dodecanol	1.0–9.7 µg/L	41–105%	[23]
9 steroids	HF-SEBLLME	milk	10 mL	Ethyl acetate	0.07–0.19 μg/L	93.6–104.6%	[28]
Progesterone, prednisolone, estradiol	MEPS	milk	5 mL	Polythiophene	16 μg/L	88.29–98.68%	[35]
***NSAIDs***							
Indomethacin, flufenamic acid, nimesulide, phenylbutazone	DLLME	water	5 mL	D-limonene	0.36–2.69 µg/L	80.99–104.92%	[24]
Diclofenac, ibuprofen ketoprofen, naproxen, E2, EE2, E3	DLLME-SFO	wastewater	10	1-undecanol	0.22–1.29 µg/L	59.3–92.5	[25]
Etodolac, naproxen, ketoprofen, diclofenac, flurbiprofen	DLLME-FASS-CE	milk, dairy products	2.0 g	Chloroform	10.0–43.7 μg/kg	77.4–109.3%	[26]
Ibuprofen, diclofenac, oxaprozin, salicylic acid	UA-HDES-DLLME	water, milk	30 mL	HDES	1–5 μg/L	79.42–107.52%	[27]
Diclofenac, mefenamic acid, flurbiprofen, ketoprofen	LLME-DES	milk	10 g	Menthol	0.01–0.03 μg/kg	82–91%	[29]
Diclofenac, ibuprofen, ketoprofen, naproxen, E1, E2, E3, EE2	DLLME-SFO	milk, egg	10 mL, 3 g	1-undecanol	2.29–5.46 µg/L 7.38–18.65 µg/kg	74.49–108.6%	This work

HF-SEBLLME—hollow fiber-based stirring extraction bar liquid–liquid microextraction; MEPS—microextraction in packed sorbent; DLLME-FASS-CE—dispersive liquid–liquid microextraction coupled with field-amplified sample stacking in capillary electrophoresis; LLME-DES—liquid–liquid microextraction with deep eutectic solvent; UA-HDES-DLLME—ultrasound-assisted-hydrophobic deep eutectic solvents with dispersive liquid–liquid microextraction.

**Table 5:** The minimum, central point, and maximum value of the variables used for experimental design modeling.

Variables	Values		
	Lowest (−1)	Central Point (0)	Highest (+1)
NaCl (mg) (X1)	0	250	500
Disperser (µL) (X2)	50	175	300
Extractant (µL) (X3)	40	70	100
pH (X4)	2	5	7

## Data Availability

Data are contained within the article and Supplementary Materials.

## Supplementary Materials

The following supporting information can be downloaded at: https://www.mdpi.com/article/10.3390/molecules29010096/s1, Table S1: The set code value of variables used in each experiment and the values of extraction recovery (%) obtained for each compound; Table S2: The magnitude of the effects and the significance level for the variables and their interactions; Table S3: Results obtained for contaminated milk and egg samples; Table S4: Results of steroids and NSAIDs obtained on the milk analyzed samples; Table S5: Results of steroids and NSAIDs obtained on the milk analyzed samples. Table S6. Physical-chemical properties of tested compounds and abbreviations; Figure S1: The optimal conditions predicted by model, minimum, maximum and optimum extraction recovery values, and the value of desirability function; Figure S2: Map indication of all 15 sampling points in the investigated area.
