# Supplementary material for: Simultaneous Determination of Steroids and NSAIDs, Using DLLME-SFO Extraction and HPLC Analysis, in Milk and Eggs Collected from Rural Roma Communities in Transylvania, Romania
Source: Molecules. 2023 Dec 22;29(1):96. doi: 10.3390/molecules29010096 (PMC10780084; doi:10.3390/molecules29010096)
Supplement: Supplementary file 1 [file molecules-29-00096-s001.zip › molecules-2775382-supplementary.pdf]

SUPPLEMENTARY INFORMATION

Article

Simultaneous Determination of Steroids and NSAIDs, Using DLLME-SFO Extraction and HPLC Analysis, in Milk and Eggs Collected from Rural Roma Communities in Transylvania, Romania

Mihaela Cătălina Herghelegiu [1], Vlad Alexandru Pănescu [1], Victor Bocoș-Binţinţan [1,*], Radu-Tudor Coman [2], Vidar Berg [3], Jan Ludvig Lyche [3], Maria Concetta Bruzzoniti [4] and Mihail Simion Beldean-Galea [1,*]

1 Faculty of Environmental Science and Engineering, Babeș-Bolyai University, 1 Kogălniceanu Str., 400084 Cluj-Napoca, Romania
2 Faculty of Medicine, "Iuliu Hațieganu" University of Medicine and Pharmacy, 8 Babeș Str., 400012 Cluj-Napoca, Romania
3 Faculty of Veterinary Medicine, Norwegian University of Life Sciences, 1433 Ås-Oslo, Norway
4 Department of Chemistry, University of Turin, via P. Giuria 5, 10125 Turin, Italy; mariaconcetta.bruzzoniti@unito.it
* Correspondence: victor.bocos@ubbcluj.ro (V.B.-B.); simion.beldean@ubbcluj.ro (M.S.B.-G.)

The supplementary information contains 10 pages, and includes 6 tables and 2 figures.

List of contents of the supplementary information:

Table S1. The set code value of variables used in each experiment and the values of extraction recovery (%) obtained for each compound.
Table S2. The magnitude of the effects and the significance level for the variables and their interactions.
Table S3. Results obtained for contaminated milk and egg samples.
Table S4. Results of steroids and NSAIDs obtained on the milk analyzed samples.
Table S5. Results of steroids and NSAIDs obtained on the milk analyzed samples.
Table S6. Physical-chemical properties of tested compounds and abbreviations.

Figure S1. The optimal conditions predicted by model, minimum, maximum and optimum extraction recovery values, and the value of desirability function.
Figure S2. Map indication of all 15 sampling points in the investigated area.

Table S1. The set code value of variables used in each experiment and the values of extraction recovery (%) obtained for each compound.

Exp. no	Salt (X1)	Disperser (X2)	Extractant (X3)	pH (X4)	HCOR	E1	E3	EE2	E2	KET	NAP	IBU	DIC
1	1	-1	-1	-1	12.3	42.9	52.6	59.4	61.9	33.4	34.5	57.3	57.1
2	1	-1	1	-1	25.3	68.6	77.6	87.8	92.9	84.0	89.0	91.0	92.1
3	1	1	-1	-1	19.1	47.1	56.3	69.2	74.3	46.1	50.3	64.5	63.8
4	1	1	1	-1	23.4	68.5	65.2	91.8	96.4	91.1	96.0	94.1	93.0
5	-1	-1	-1	-1	11.5	30.1	58.2	71.1	75.7	39.1	50.0	59.6	54.6
6	-1	-1	1	-1	13.9	46.6	66.7	86.0	87.4	42.0	41.3	73.9	76.0
7	-1	1	-1	-1	9.5	26.7	54.9	63.9	68.9	26.0	29.6	59.1	51.9
8	-1	1	1	-1	18.3	53.4	51.2	89.1	90.0	53.1	54.3	78.2	81.4
9	-1	-1	-1	1	19.7	49.5	29.0	60.4	61.8	49.5	59.7	62.8	63.4
10	-1	-1	1	1	20.6	72.4	55.1	88.8	84.8	90.0	94.0	93.0	91.0
11	-1	1	-1	1	14.7	44.6	24.9	67.7	68.8	44.6	54.7	67.7	70.3
12	-1	1	1	1	21.6	73.3	53.9	74.0	76.2	73.3	71.6	75.4	78.8
13	1	-1	-1	1	15.1	59.2	42.4	71.2	68.0	59.2	65.1	69.2	66.1
14	1	-1	1	1	28.8	78.3	65.4	85.3	81.1	78.3	78.8	79.5	78.0
15	1	1	-1	1	20.1	57.8	40.0	69.3	70.0	57.8	63.1	68.7	68.3
16	1	1	1	1	25.5	85.2	68.8	83.0	82.0	85.2	81.5	81.1	81.2
17	0	0	0	0	27.7	77.9	68.1	71.4	60.5	82.2	86.1	81.8	83.4
18	0	0	0	0	21.8	70.2	69.5	71.8	61.5	81.6	87.2	83.1	84.3
19	0	0	0	0	21.3	77.1	68.9	72.2	61.2	82.9	85.7	82.6	82.8

Table S2. The magnitude of the effects and the significance level for the variables and their interactions.

Source	Logworth		PValue	
Extractant	2.626		0.00237	
Salt*Disperser*Extractant*pH	1.008		0.09824	
pH	0.840		0.14446	^
Salt	0.793		0.16098	^
Extractant*pH	0.768		0.17046	^
Disperser*Extractant*pH	0.724		0.18865	^
Salt*Disperser*Extractant	0.577		0.26460	^
Salt*Extractant*pH	0.569		0.26985	^
Disperser*pH	0.566		0.27171	^
Salt*pH	0.562		0.27410	^
Salt*Disperser	0.560		0.27534	^
Disperser*Extractant	0.468		0.34066	^
Salt*Disperser*pH	0.370		0.42697	^
Salt*Extractant	0.321		0.47723	^
Disperser	0.220		0.60312	^

Figure S1. The optimal conditions predicted by model, minimum, maximum and optimum extraction recovery values, and the value of desirability function.

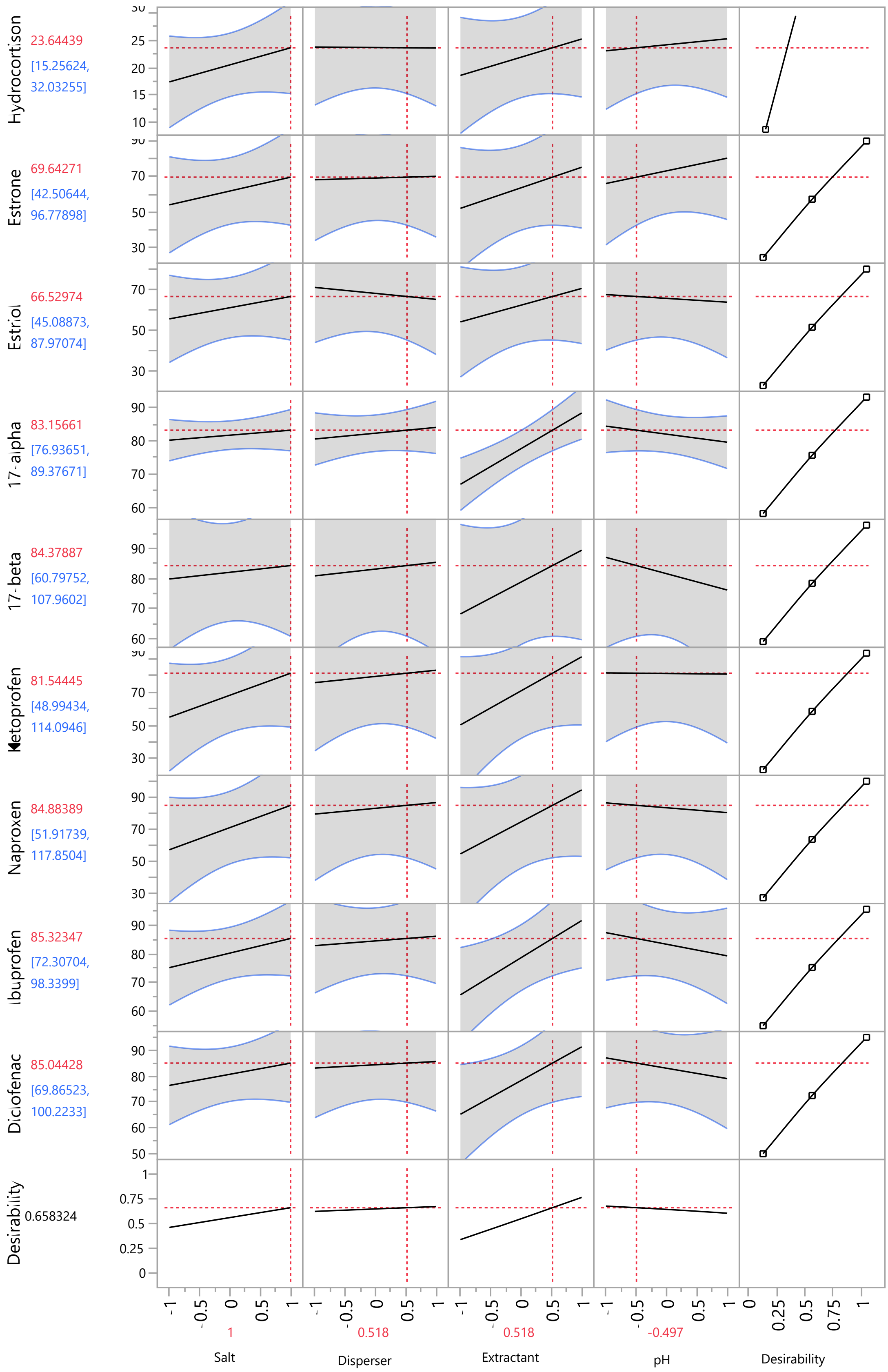

Table S3. Results obtained for contaminated milk and egg samples.

Samples/ compounds	Amount found in samples [ng]								
	HCOR	E1	E2	EE2	E3	KET	NAP	DIC	IBU
Milk initial	nd	0.11	0.06	1.18	0.08	nd	0.40	0.07	0.30
Milk spiked 1	10.83	86.72	89.50	81.40	63.46	76.80	74.70	74.46	87.07
Milk spiked 2	9.98	85.46	87.61	82.91	63.29	77.89	74.02	75.12	88.21
Milk spiked 3	10.60	86.01	87.68	84.84	64.2	78.89	74.49	74.17	87.92
Average milk	**10.47**	**86.06**	**88.26**	**83.05**	**63.32**	**77.86**	**74.40**	**74.58**	**87.73**
Standard deviation	0.44	0.63	1.07	1.72	0.13	1.04	0.34	0.48	0.59
Egg initial	nd	0.28	0.26	nd	0.35	0.17	0.82	0.12	1.57
Egg spiked 1	10.66	85.85	92.20	90.87	63.25	76.05	77.91	78.34	95.07
Egg spiked 2	9.77	81.61	87.66	86.98	65.87	76.71	75.68	76.85	92.02
Egg spiked 3	10.5	82.73	89.9	88.83	63.74	75.48	76.5	77.7	94.54
Average Egg	**10.31**	**83.40**	**89.92**	**88.89**	64.29	**76.08**	**76.70**	**77.63**	**93.88**
Standard deviation	0.47	2.20	2.27	1.95	1.39	0.62	1.13	0.75	1.63

"nd" – not detected

Table S4. Results of steroids and NSAIDs obtained on the milk analyzed samples.

Sampling point	Concentration [µg/L]							
	E1	E2	EE2	E3	KET	NAP	DIC	IBU
Almașu	0.97	<MDL	<MDL	<MDL	<MDL	<MDL	<MDL	<MDL
Avrămești	0.85	1.10	0.87	<MDL	1.03	1.23	0.78	2.18
Băgaciu	<MDL	<MDL	<MDL	<MDL	<MDL	<MDL	<MDL	<MDL
Coltău	<MDL	<MDL	<MDL	<MDL	<MDL	<MDL	<MDL	3.41
Delenii	1.59	<MDL	2.54	0.77	0.90	<MDL	0.71	1.15
Diosig	<MDL	<MDL	<MDL	<MDL	<MDL	<MDL	<MDL	<MDL
Gornești	<MDL	<MDL	1.31	<MDL	<MDL	<MDL	<MDL	<MDL
Gurghiu	<MDL	<MDL	1.86	<MDL	<MDL	<MDL	<MDL	<MDL
Racovița	<MDL	1.62	4.30	0.95	<MDL	<MDL	<MDL	<MDL
Roșia Montana	<MDL	<MDL	0.67	<MDL	<MDL	<MDL	<MDL	<MDL
Saschiz	<MDL	<MDL	1.32	<MDL	<MDL	<MDL	<MDL	<MDL
Șaeș	<MDL	1.92	3.49	<MDL	<MDL	<MDL	<MDL	2.90
Terebești	<MDL	<MDL	<MDL	<MDL	<MDL	<MDL	<MDL	2.66
Turulung	<MDL	<MDL	<MDL	<MDL	<MDL	<MDL	0.77	1.76
Țețchea	<MDL	<MDL	<MDL	0.86	<MDL	<MDL	<MDL	0.81

MDL- method detection limit

Table S5. Results of steroids and NSAIDs obtained on the egg analyzed samples.

Sample point	Concentration [μg/kg]							
	E1	E2	EE2	E3	KET	NAP	DIC	IBU
Almașu	<MDL	<MDL	<MDL	0.98	0.81	<MDL	<MDL	0.89
Avrămești	<MDL	1.42	<MDL	<MDL	0.98	1.63	<MDL	2.20
Băgaciu	0.73	2.79	2.23	<MDL	<MDL	2.10	<MDL	<MDL
Coltău	<MDL	2.69	2.04	<MDL	1.99	1.68	<MDL	2.28
Delenii	<MDL	2.64	<MDL	1.08	<MDL	<MDL	<MDL	2.11
Diosig	<MDL	<MDL	<MDL	1.16	<MDL	1.26	<MDL	<MDL
Gornești	<MDL	2.79	2.19	1.99	<MDL	2.10	<MDL	1.76
Gurghiu	<MDL	<MDL	<MDL	<MDL	<MDL	<MDL	<MDL	1.44
Racovița	<MDL	<MDL	<MDL	<MDL	<MDL	<MDL	<MDL	<MDL
Roșia Montana	<MDL	<MDL	0.89	<MDL	0.78	<MDL	<MDL	<MDL
Saschiz	<MDL	<MDL	<MDL	1.01	<MDL	<MDL	<MDL	<MDL
Șaeș	<MDL	<MDL	<MDL	<MDL	1.28	1.71	<MDL	<MDL
Terebești	<MDL	<MDL	<MDL	0.89	<MDL	<MDL	<MDL	0.98
Turulung	<MDL	<MDL	<MDL	<MDL	0.89	1.72	<MDL	1.62
Țețchea	<MDL	<MDL	<MDL	0.98	<MDL	<MDL	<MDL	<MDL

MDL- method detection limit

Table S6. Physical-chemical properties of tested compounds and abbreviations

Compound name	Molecular formula	Abbreviation	LogP	pKa
Ibuprofen	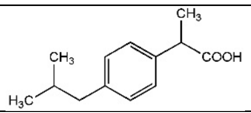	IBU	3.97	5.03
Diclofenac	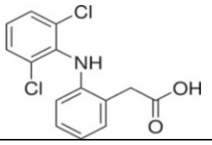	DIC	4.51	4.15
Ketoprofen	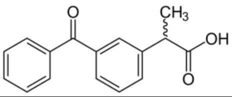	KET	3.12	4.45
Naproxen	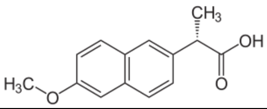	NAP	3.18	4.15
Hydrocortisone	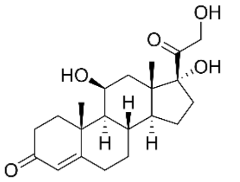	HCOR	1.61	12.59
Estrone	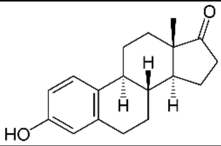	E1	2.6	10.77
17β-estradiole	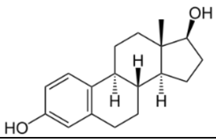	E2	4.01	10.08
17α-ethynilestradiole	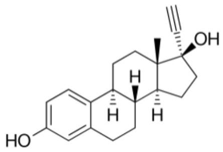	EE2	3.67	10.21
Estriol	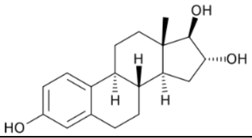	E3	2.45	10.54

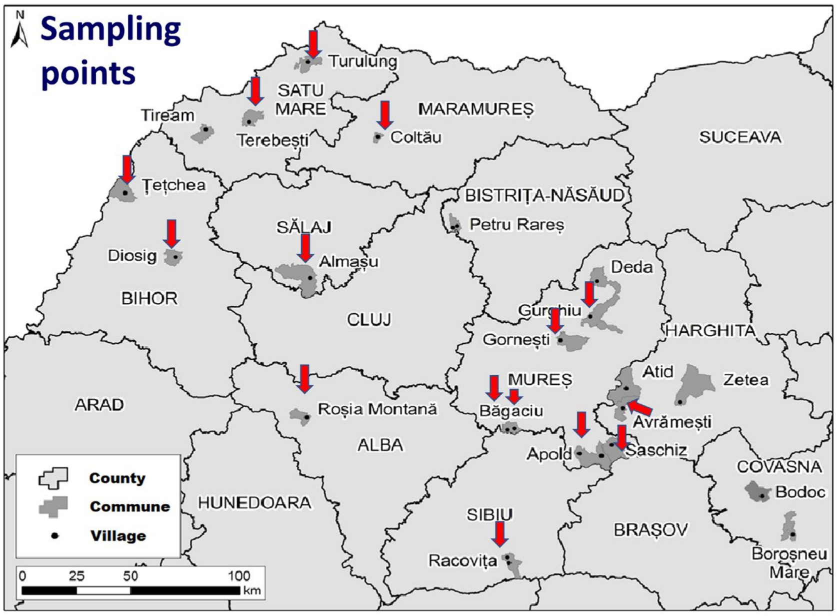

Figure S2. Map indication of all 15 sampling points in the investigated area
